# GlucoTRIG: a novel tool to determine the nutritional quality of foods and meals in general population

**DOI:** 10.1186/s12944-020-01268-w

**Published:** 2020-05-04

**Authors:** Rohith N. Thota, Paul J. Moughan, Harjinder Singh, Manohar L. Garg

**Affiliations:** 1 Nutraceuticals Research Program, School of Biomedical Sciences & Pharmacy, University of Newcastle, Callaghan, NSW Australia; 2 Riddet Institute, Massey University, Palmerston North, New Zealand; 3 Priority Research Centre in Physical Activity & Nutrition, University of Newcastle, University of Newcastle, Callaghan, NSW 2308 Australia

**Keywords:** Composite meals, Ranking criteria, GlucoTRIG, Insulin, Triglycerides, Macronutrients, Nutrient profiling, Diet, Metabolic disease

## Abstract

**Background:**

This study aimed to develop a novel criterion, GlucoTRIG, to rank meals for healthiness, that considers both glycaemic (serum insulin) and lipaemic (serum triglycerides) responses.

**Methods:**

Healthy volunteers (*n* = 10) were recruited with the aim of deriving a standard GlucoTRIG value for a reference meal. Volunteers consumed the reference meal (2 regular slices of wholemeal bread; 250 mL chocolate flavoured milk; 7 g butter and 11 g peanut butter) comprising of carbohydrate, fat and protein (41, 40 and 16% energy respectively) on three different occasions with a minimum washout period of 3 days. The GlucoTRIG value was determined as the difference between the product of insulin and triglyceride obtained from venous blood samples at baseline and the product of insulin and triglyceride at 180 min.

**Results:**

There were no significant differences in the participants’ dietary intakes and their metabolic parameters between three visits (*P* > 0.005). The GlucoTRIG value obtained from three mean values of the reference meal was found to be 19 ± 3.5. There were no significant (*P* = 0.2303) differences observed between the GlucoTRIG values for the three visits.

**Conclusion:**

GlucoTRIG, consisting of both glycaemic and lipaemic responses, may be a physiologically relevant tool to rank foods and meals for reducing the risk of metabolic diseases.

**Trial registration:**

ACTRN12619000973112.

## Background

Regular consumption of low glycaemic index (GI) foods, minimizing spikes in blood sugar and insulin levels, is associated with a reduced risk for developing type 2 diabetes and heart disease, and assists in weight management [1, 2]. Consequently, the GI and the glycaemic load are frequently used to grade foods and meals as determinants of their healthiness, and such measures have made a major contribution to understanding the link between food composition and metabolic outcomes [2]. Single foods, however, are rarely consumed in isolation, rather meals comprising a mix of macronutrients make up normal diets. The metabolic response to foods, meals and diets differ depending upon the relative proportions of the macronutrients [3]. The synergism and interactions among the macronutrients together with effects of processing and preparation of foods (overall food matrix) cannot be captured by a single nutrient response. Further, that the GI of a food or meal can be altered by addition of fat, remains a major limitation of GI as an overall index of the healthiness of foods and meals [4]. Fat enrichment may lower the GI of a food/meal, but this does not make the meal more healthy, as it can be associated with postprandial lipaemia and an increased inflammatory response [5]. Moreover, the GI methodology does not permit testing for healthiness of foods and meals with little or no carbohydrate [6]. It cannot provide a guide to the relative insulin response of many foods. Although carbohydrate is the primary stimulus for insulin secretion, protein and fat rich foods also elicit a significant insulin response and, when combined with carbohydrate, may significantly increase the postprandial glycaemic and lipaemic responses. It has been widely assumed that the insulin response is proportional to blood glucose levels, therefore GI can predict the response to insulin. However, protein- and fat-rich foods may induce substantial insulin secretion despite producing relatively small blood glucose responses [7–9]. Categorising foods and meals based on a physiological response to both glucose and fat could be an effective approach in promoting health, and improving the understanding of consumers to identify foods that are healthier and prevent diet related diseases [10]. Foods with low postprandial glycaemic and lipaemic responses may have the potential in assisting in the prevention of weight gain and the further development of chronic metabolic diseases.

As humans are in an absorptive state (non-fasting) for over 18 h in a day, nutrient profiling models that capture postprandial glycaemic and lipaemic responses could be of both practical and clinical significance in preventing chronic diet related disease conditions. Recent research [5, 11] have recently highlighted the necessity of factoring in post-prandial triglyceride response for determining the healthiness of foods and meals. Adding a large amount of fat to carbohydrate rich meals can reduce the glucose response [12], but it also increases the insulin response [13] and inflammation [14]. Though there is consistent evidence that high dietary fat intake in general is associated with both insulin resistance and post-prandial metabolic abnormalities [5], the quantity and quality of fat is mostly neglected when assessing the overall healthiness of foods and meals.

Consumption of meals rich in fat and processed sugars results in transient elevation of both blood glucose levels and lipids, and experiencing these exaggerated postprandial glycaemic and lipaemic responses several times during a 24-h period, represents a disruption of the homeostasis of metabolic pathways [15, 16]. Therefore hypothesis of the study is built on a criterion based on postprandial serum insulin and triglyceride responses to the macronutrient content of meals could provide an enhanced understanding of the healthiness of foods and meals. The aim of the study, therefore, was to describe the basis of GlucoTRIG, as a means of ranking foods based on their physiological fasting and postprandial serum insulin and triglyceride response over a period of 3 h, and to determine the GlucoTRIG value for a reference meal which could be used in future studies as a comparator for testing meals in general.

## Methods

Twelve healthy volunteers, both males and females, were screened using telephone interviews (University Of Newcastle, New South Wales, Australia) between September and November 2018. All subjects met the inclusion criteria: aged between 18 and 40 years at initial assessment with body mass index (BMI) between 18 and 29.9 Kg/m^2^; non-smokers; not pregnant; currently not taking any lipid lowering drugs or supplements (e.g. statins, fish oil) or anti-hypertensive drugs; no history of eating disorders, congestive heart failure, stroke, myocardial infarction, coronary artery bypass graft, or atherosclerotic cardiovascular disease, gastrointestinal disorder or liver disease; no allergy or intolerance to any of the food products or ingredients used in the study. The study protocol was approved by the University of Newcastle Human Research Ethics Committee (H−2016-0315). This work has been carried out in accordance with the Code of Ethics of the World Medical Association (Declaration of Helsinki). All participants gave written informed consent. The study was registered on Australian New Zealand Clinical Trials Registry (ANZCTR) under study number ACTRN12619000973112.

### Reference meal

The reference meal consisted of wholemeal bread (2 regular slices consisting of Wheat Flour, Water, Wheat Flour, Wheat Gluten, Yeast, Vinegar, Iodised Salt, Canola Oil); 28 g each and manufactured by Woolworths select, Newcastle, Australia), chocolate flavoured milk (250 mL; consisting of whole milk, skim milk, sucrose, milk solids, cocoa powder, maltodextrin and manufactured by OAK Parlamat, Newcastle, Australia), unsalted butter (7 g) and peanut butter (11 g; consisting of roasted peanuts, vegetable oil, sugar and salt and manufactured by Kraft Foods group Inc. Melbourne, Australia) delivering carbohydrates (41%), fat (40%) and protein (16%) of the total energy content (⁓2000kj) of the meal. The detailed nutrient composition of the reference meal is given in Table 1.

**Table 1:** Description of macronutrient composition of the reference meal

Food category	Serving size	Carbohydrate	Sugar	Starch	Total fat	Saturated fat	Polyunsaturated fat	Monounsaturated fat	Dietary fibre	Protein
	(g)	(g)	(g)	(g)	(g)	(g)	(g)	(g)	(g)	(g)
**Reference meal** Wholemeal bread^a^ Butter^b^ Peanut butter^c^ Chocolate milk^d^	*339*	*50*	*26*	*24*	*22*	*11*	*1*	*8*	*6*	*19*
% energy		*41*			*40*				*2*	*16*

^a^2 slices (28 g each), Woolworths select; ^*b*^*7 g portion, Western Star;*^*c*^*11g portion, Kraft smooth peanut butter;*^*d*^*250mL OAK chocolate milk, Parmalat;*

### Study design and test day protocol

Volunteers received the reference meal on 3 separate days with a wash out period of at-least 3 days between each study day. Following screening, eligible subjects were advised to refrain from any vigorous physical activity and alcohol intake 24 h before the test day and asked not to consume a high calorie (rich in carbohydrates and fat) meal the night before the test day. Participants’ dietary information was obtained through 24-h food recall and processed through FoodWorks Version: 8.0.3551 (Xyris Software (Australia) Pty Ltd) to check whether their dietary energy and macronutrient intakes remained unchanged during the study period. Physical activity of the participants was assessed on the first test day using the International Physical Activity Questionnaire (long form) designed to capture the frequency, duration and intensity of physical activity undertaken during the previous 7 days. Participants were categorised as having a low physical activity level if they did not meet moderate (5 or more days of moderate intensity activity and/or walking of at least 30 min per day or 5 or more days of any combination of walking, moderate intensity activities achieving a minimum total physical activity of at least 600 metabolic equivalents per min/week) or high (vigorous-intensity activity on at least 3 days achieving a minimum total physical activity of at least 1500 metabolic equivalents per min/week or 7 days of any combination of activities achieving a minimum total physical activity of at least 3000 metabolic equivalents per min/week) criteria. On the test morning, subjects reported to the laboratory after an overnight fast of at latest 12 h. Participants were asked to consume the reference meal within 20 min. No other food or drink was allowed during the 3 h period on the test days.

### Outcome measures

After the participant’s arrival at the Nutraceutical Research Program clinical trial facility at the University of New Castle on the first test day in fasting state, medical history, demographics, BMI were recorded. Body composition was determined using bioelectrical impedance (InBody 230, Biospace Co., Ltd. Seoul, Korea). Venous blood samples were collected by an in house certified phlebotomist at 0 min and 180 min. Fasting and postprandial serum insulin (pmol/L), triglycerides (mmol/L) and glucose (mmol/L) were then measured by using standard laboratory procedures (Hunter Area Pathology Service).

Determination of GlucoTRIG

The GlucoTRIG value was calculated using the formula:

$$ \left({\mathrm{Triglycerides}}_{180\mathrm{min}}\ast {\mathrm{Insulin}}_{180\mathrm{min}}\right)-\left({\mathrm{Triglycerides}}_{0\mathrm{min}}\ast {\mathrm{Insulin}}_{0\mathrm{min}}\right) $$

### Statistical analysis

Data were expressed as mean ± SEM and number (n) for categorical variables. Normality of the data were assessed using histograms and the Shapiro-Wilk test. Between visits comparisons were made using a repeated measures analysis of variance (ANOVA), and the Bonferroni post hoc test for multiple comparisons. For all the statistical analysis, *P* values less than 0.05 were considered to be significant. Statistical analysis was performed using the STATA version 14.1 (StataCorp, Texas, USA).

## Results

**Fig. 1:**
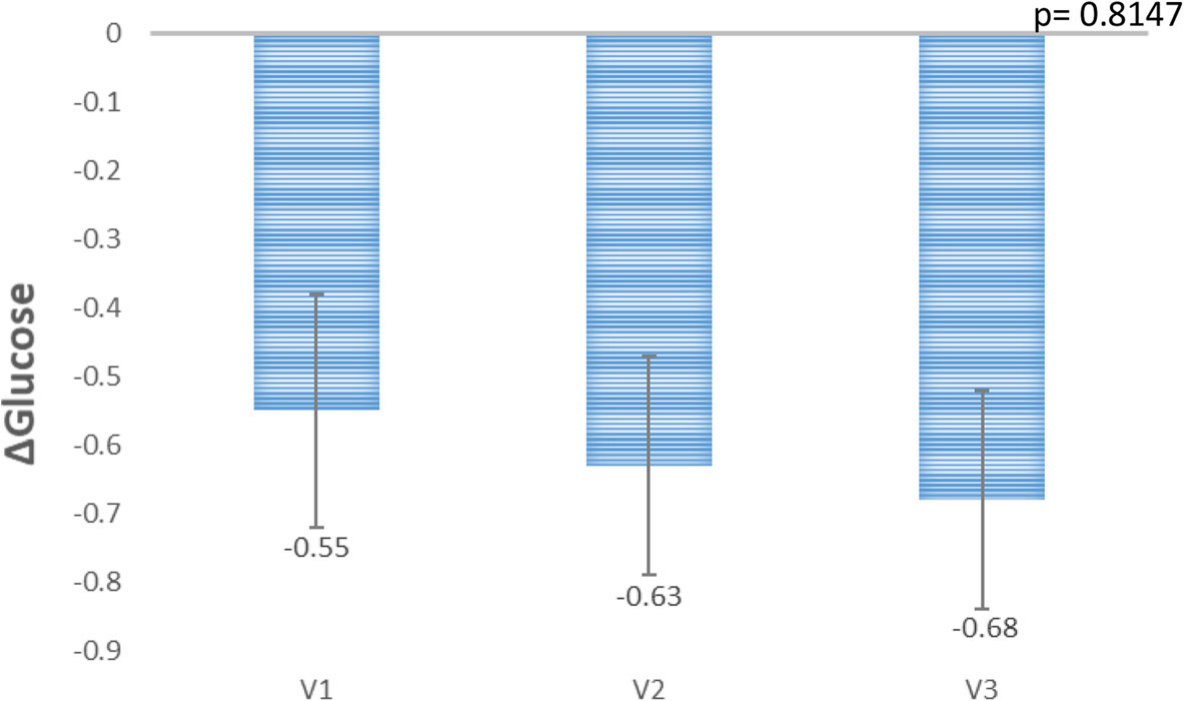
Mean change in the plasma glucose (180–0 min) values for the refernce meal on visits 1 (V1), 2(V2) and 3(V3). Data presented as mean ± SEM (*n* = 10). *P*-value is derived from the repeated measures ANOVA

**Fig. 2:**
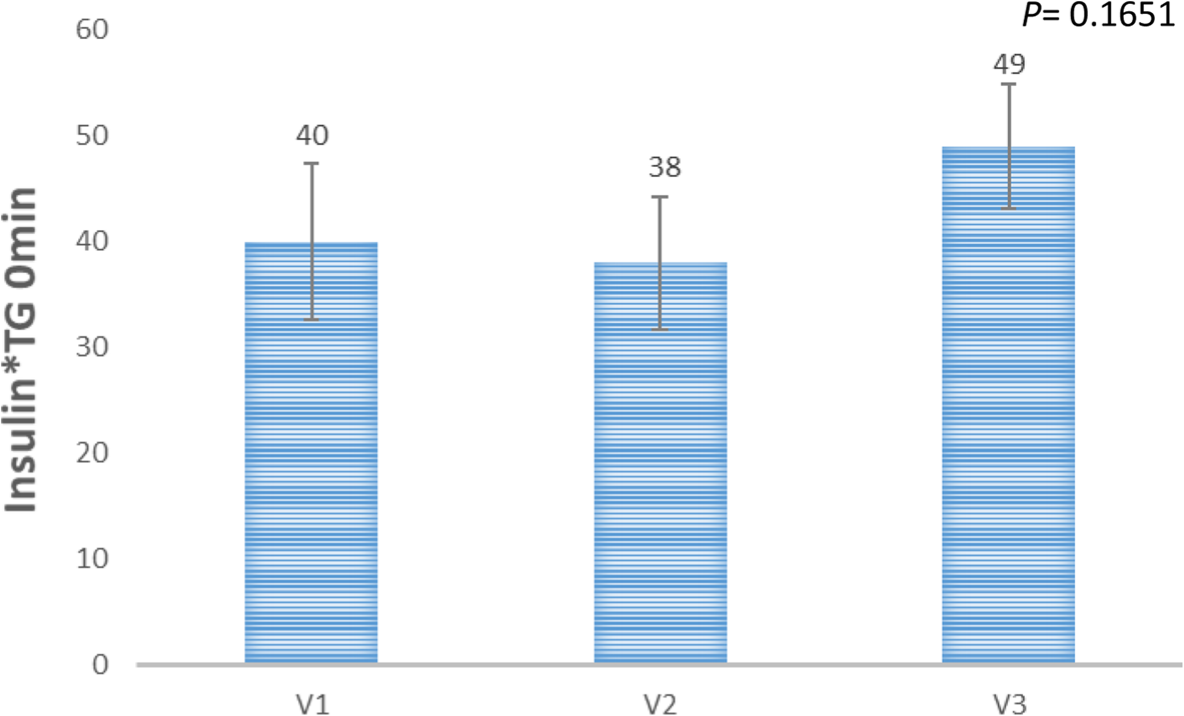
Insulin*Triglyceride (0 min) values for the refernce meal on visits 1 (V1), 2(V2), 3(V3). Data presented as mean ± SEM (*n* = 10). *P-*value is derived from the repeated measures ANOVA

**Fig. 3:**
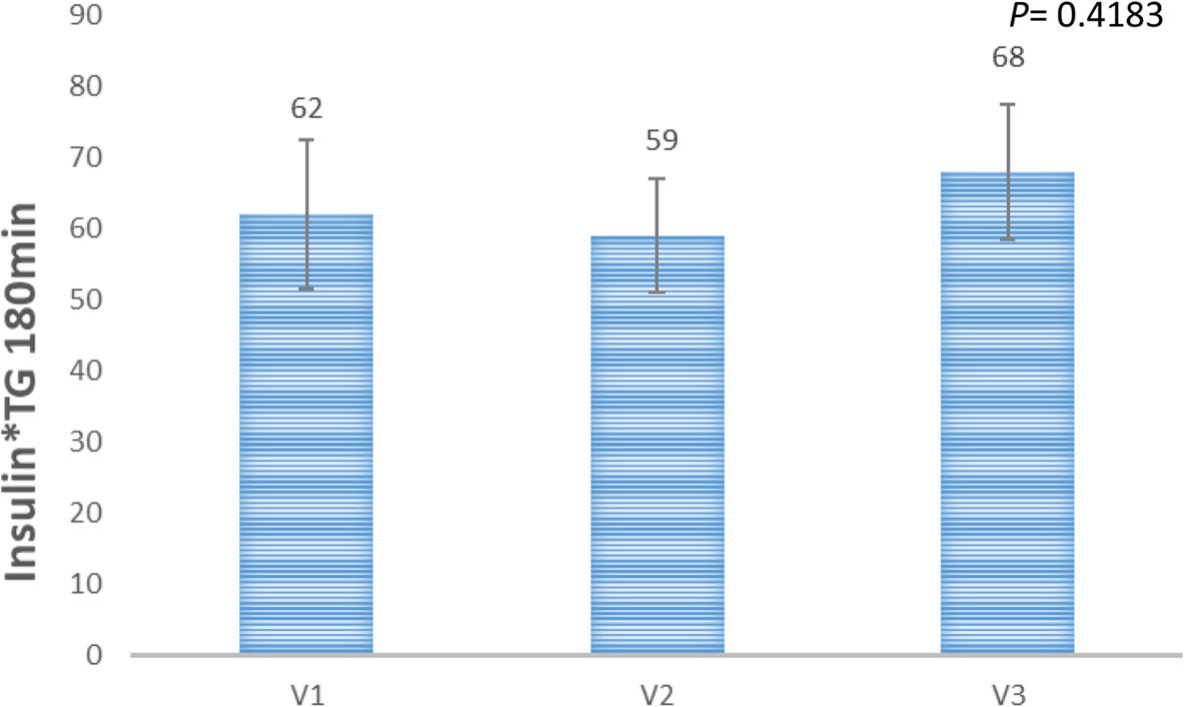
Insulin*Triglyceride (180 min) values for the refernce meal on visits 1 (V1), 2(V2), 3(V3). Data presented as mean ± SEM (*n* = 10). *P*-value is derived from the repeated measures ANOVA

Two of the 12 recruited subjects recruited were excluded as outliers based on the abnormal pathological reports and are excluded from the final analysis using standard outlier rule (mean ± 2 x SD) of the GlucoTRIG values. Ten healthy subjects (both females and males) aged 27.7 ± 1.2 years with BMI of 23.4 ± 0.4 kg/m^2^ were included in the final analysis (Table 2). Fasting serum glucose (4.4 ± 0.1 mmol/L), insulin (44.1 ± 4.4 pmol/L) and triglycerides (0.8 ± 0.1 mmol/L) concentrations were also in healthy range (Table 2). Physical activity analysis indicated seven participants with low physical activity and three with moderate physical activity levels at the baseline visit. There were no significant changes observed in the macronutrient (carbohydrate, protein, total fat) and fibre intake for the 24 h period before each test day (Table 3). In addition, no sex based significant differences were observed in the macronutrient intake (carbohydrate, protein and total fat), fibre and energy intake levels (Table 4) The mean change in the serum glucose responses to the reference meal did not vary (*P* = 0.8147) between the visits (Fig. 1). Mean (Triglycerides_180min_ * Insulin_180min_) and (Triglycerides_0min_ * Insulin_0min_) for the reference food were not statistically significantly (*P* > 0.05) different between the three visits (Figs. 2 and 3).

**Table 2:** Baseline characteristics of the participants

Participant characteristics	All Participants (***n*** = 10) Mean ± SEM values
**Age**	27.7 ± 1.2
**Males/Females (n/n)**	3/7
**Weight (kg)**	67 ± 1.8
**Body mass Index (kg/m**^**2**^**)**	23 ± 0.4
**Fasting glucose (mmol/L)** **(normal range - 4.0-5.4 mmol/L)**	4.4 ± 0.14
**Fasting insulin (mU/L)** **(normal range - < 10 mu/L)**	6.1 ± 0.62
**Fasting triglycerides (mmol/L)** **(normal range - < 1.7 mmol/L)**	0.8 ± 0.1
**Physical activity**	Low (7) Medium 3)

Values are reported as means ± SEM. n represents the number of subjects. Physical activity of the participants is measured and categorised using International Physical Questionnaire (IPAQ) –long form

**Table 3:** Macronutrient intakes of the participants for the 24 h period before the test days

	Reference meal		
Macronutrients	Visit 1	Visit 2	Visit 3
	*N* = 10	*N* = 10	*N* = 10
**Energy (Kj)**	7778 ± 503	7459 ± 507	7151 ± 620
**Carbohydrate (g)**	190 ± 20.9	174 ± 18.6	176 ± 23.1
**Sugars (g)**	62 ± 10.5	66 ± 11.7	52 ± 9.9
**Starch (g)**	129 ± 20.1	108 ± 12.8	125 ± 14.9
**Total fat (g)**	76 ± 6.9	78 ± 7.3	72 ± 10.0
**Saturated fat (g)**	22 ± 2.9	27 ± 2.9	26 ± 4.8
**Polyunsaturated fat (g)**	12 ± 1.7	12 ± 1.7	10 ± 1.1
**Monounsaturated fat (g)**	33 ± 3.4	33 ± 3.2	29.1 ± 4.6
**Cholesterol (g)**	301 ± 67.8	211 ± 39.8	247 ± 53.4
**Dietary fibre (g)**	21 ± 2.0	21 ± 2.1	23 ± 3.2
**Protein (g)**	92 ± 7.7	86 ± 12.6	79 ± 6.2

Values are reported as means ± SEM. N represents the number of subjects

**Table 4:** Macronutrient intakes of the males and females for the 24 h period before the test days

	Males			Females		
Macronutrients	Visit 1	Visit 2	Visit 3	Visit 1	Visit 2	Visit 3
Energy (kj)	6341 ± 848.5	7405 ± 1659.1	5798 ± 436.0	8394 ± 479.7	7482 ± 1051.9	7731 ± 780.8
Carbohydrate (g)	155 ± 34.9	117 ± 25.3	120 ± 27.7	205 ± 25.4	199 ± 47.2	201 ± 26.6
Sugars (g)	51 ± 12.4	44 ± 6.5	42 ± 11.5	66 ± 14.2	75 ± 7.4	56 ± 13.5
Starch (g)	105 ± 39.6	74 ± 19.3	79 ± 17.1	139 ± 24.1	123 ± 13.5	145 ± 14.7
Total fat (g)	61.6 ± 10.7	93 ± 15.9	59 ± 11.2	82 ± 8.0	72 ± 7.4	77 ± 13.5
Saturated fat (g)	21 ± 5.5	30 ± 3.9	17 ± 4.6	23 ± 3.7	26 ± 4.0	30 ± 6.3
Polyunsaturated fat (g)	9 ± 0.5	15 ± 4.6	8 ± 1.8	14 ± 2.2	10 ± 1.4	11 ± 1.4
Monounsaturated fat (g)	27 ± 4.3	41 ± 6.2	28 ± 6.6	35.5 ± 4.3	30 ± 3.3	30 ± 6.4
Cholesterol (g)	286 ± 52.8	331 ± 76.5	455 ± 57.5	307 ± 97.1	159 ± 32.7	157 ± 34. 4
Dietary fibre (g)	16 ± 1.2	20 ± 5.6	17 ± 3.5	24 ± 2.4	21 ± 2.3	25 ± 4.1
Protein (g)	76.7 ± 5.2	105 ± 33.7	87 ± 19.5	98.4 ± 9.9	77 ± 11.6	75 ± 4.5

Values are reported as means ± SEM for males (*n* = 3) and females (*n* = 7)

### GlucoTRIG values of the reference meal

**Fig. 4:**
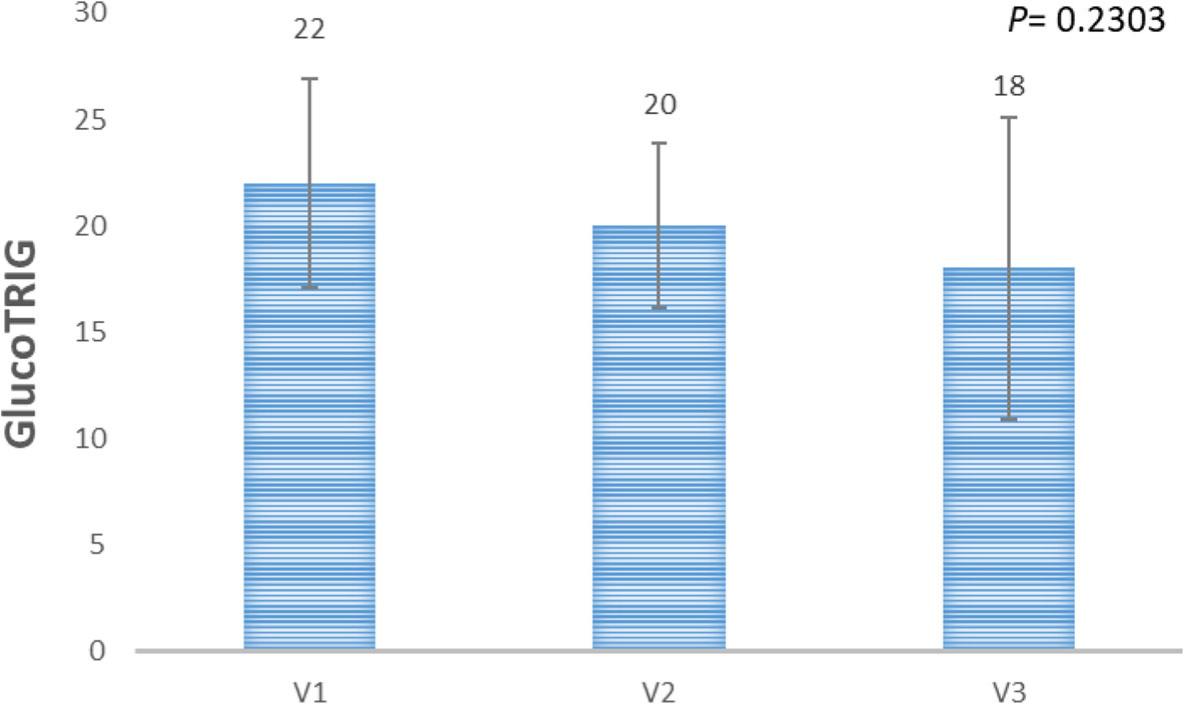
Mean GlucoTRIG value for the reference meal on Visits 1 (V1), 2(V2), 3(V3). Data presented as mean ± SEM (*n* = 10). *P*-value is derived from the repeated measures ANOVA

A baseline GlucoTRIG value for the reference meal was obtained by repeating measures for the meal three times with a washout period of at least 3 days. The GlucoTRIG value (*n* = 10) for the reference meal (wholemeal toast, butter, peanut butter, chocolate milk) was found to be 19 ± 3.5 with a coefficient of variation of 1.1% (Fig. 4). There were no significant differences in mean GlucoTRIG (*n* = 10) responses between visits (*P =* 0.2303).

## Discussion

This study outlines a new dietary index, GlucoTRIG, based on postprandial serum insulin and triglyceride (fat) responses to meals. This classification may be useful in nutritional epidemiology and clinical applications because diets provoking high postprandial glycaemic [17, 18] and lipaemic responses [19, 20] have been shown to be associated with an increased risk of diabetes, obesity, and cardiovascular disease. GlucoTRIG is a new single indicator of nutrient profiling that could be listed on food labels for consumers to make decisions regarding the healthiness of foods and the nutritional management of diet-related metabolic diseases. The study reported here establishes a reference meal composed of equivalent carbohydrate and fat proportions (41 and 40% of the total energy) which has been shown to have acceptably low between-meal variation, and can be used in future studies as a standard comparator to rank the healthiness of foods and meals.

### Physiological basis for GlucoTRIG

Insulin is a key regulator of both glucose and lipid homeostasis and any long term disruption in its signalling or action has significant impact on the development of metabolic disease [21]. Postprandial glycaemia together with dietary patterns that induce excessive insulin secretion have been associated with weight gain and a higher risk of developing type 2 diabetes [22]. Compared to healthy adults, meal-associated insulin secretion is higher, and the return of insulin to baseline between meals is delayed in obese individuals [23] although insulin mediated glucose suppression may still be normal. In individuals without diabetes, accentuated day long incremental insulin responses to meals have also been shown to be associated with coronary heart disease [18]. Parallel to the postprandial glycaemic alterations, postprandial metabolism of dietary triglycerides in response to meals is transitory usually lasting from 6 to 8 h [24], with peak triglyceride levels appearing between 180 to 240 min post consumption of the meal [24]. The amount of fat in the diet, as well its fatty acid composition, have been demonstrated to influence postprandial triglyceride metabolism [5, 24]. Food structure and the composition of macronutrients have the potential to delay or expedite digestion and absorption of lipids; and therefore may also have an effect on the duration and intensity of the postprandial lipaemia [5]. Humans, in general are in an absorptive state (non-fasting) for over 18 h during a day and therefore, postprandial triglyceride levels are now recognised as an important risk factor for cardiovascular disease [19]. A cohort study has shown that non-fasting triglycerides are associated with incident cardiovascular events, independent of traditional cardiac risk factors, levels of other lipids, and markers of insulin resistance [25].

Dietary triglycerides are hydrolysed in the small intestine by pancreatic lipase in conjunction with bile salts to free fatty acids and monoglycerides and are then absorbed by enterocytes and transported to the endoplasmic reticulum and are re-synthesised into triglycerides [26]. Triglycerides are then packaged into chylomicron particles, which are secreted into the circulation to peripheral tissues where they undergo lipolysis [26]. Hepatic lipase plays an important role in enhancing the uptake of triglyceride-rich lipoprotein via lipolysis. Persistent elevation of serum insulin as evident in individuals with insulin resistance, interferes with insulin activity and glucose uptake and free fatty acid esterification due to impairment of insulin mediated suppression of hormone sensitive lipase [27]. As a result the increased free fatty acid lipolysis and diminished free fatty acid esterification, the free fatty acids are diverted to non-adipose tissues such as liver and muscle. Disruption in the insulin signalling pathways also leads to a decrease in clearance of very low density lipoprotein (VLDL)-triacylglycerol. However, the insulin-mediated suppression of hepatic glucose production is still normal at this stage. With progression to type 2 diabetes insulin fails to suppress hepatic glucose production yet promotes lipid synthesis [28]. Excessive storage of triacylglycerol in the adipose tissue causes hypertrophy of adipocytes; together with the disruption in insulin signalling pathways, the expanded adipose tissue increase free fatty acids efflux to insulin sensitive non-adipose tissues such as skeletal muscle [29]. These excessive amounts of free fatty acids, then compete with glucose for oxidation in skeletal muscle interfering with insulin activity and glucose uptake in skeletal muscle, collectively these interactions are the underlying cause for hypertriglyceridemia, pancreatic β-cell dysfunction, and the development of type 2 diabetes, endothelial dysfunction and cardiovascular disease [30].

### Rationale for considering serum insulin and triglycerides and combining them into a single index for GlucoTRIG

In terms of meeting energy requirements, carbohydrates (starch and simple sugars) represent a major class of nutrient and are a key component of the daily diet comprising 45–55% of the energy intake [31]. Glucose released from many of the carbohydrates is utilised by several organs of the body and, presents a metabolic challenge for the maintenance of homeostasis. To maintain homeostasis, there is a complex mechanism involving release, storage and disposal of glucose [32]. Both the amount of carbohydrates and type of carbohydrates have been shown to have a major effect on the postprandial glycaemia and insulin levels. However, the pattern of the plasma postprandial plasma glucose and insulin levels elicited by different kinds of sugars differs substantially from that elicited by starch, 2–3 h post consumption of these carbohydrates. Such differences offer a potential explanation for changes in insulin sensitivity depending on the duration of exposure of insulin to the cells that might affect insulin sensitivity through down-regulation of the biological activity of insulin [33]. Jenkins et al. [2, 34] initially conceived the concept of GI as a tool for the dietary management of type 1 diabetes by comparing 50 g portions of various carbohydrates with 50 g glucose. It was assumed to apply to high available carbohydrate foods (such as potatoes, rice, cereals etc.) [34]. As such, low-GI foods were classified as those that are digested and absorbed slowly and lead to a low glycaemic response, whereas high-GI foods are rapidly digested and absorbed and show a high glycaemic response. Subsequently, the standard against which foods are compared was changed from glucose to white bread [6]. GI has proven to be a more useful nutritional concept than the classification of carbohydrates (as simple or complex, as sugars or starches, or as available or unavailable), providing insights into the relationship between physiologic effects of carbohydrate-rich foods and complications for human health [1]. Foods and diets high in available carbohydrate content have been shown to exhibit a prolonged glycaemic response, leading to the disruption of blood glycaemia. GI has also been evaluated in several prospective observational studies which have shown that the chronic consumption of a diet with a high glycaemic load is independently associated with an increased risk of developing type 2 diabetes and cardiovascular disease [2]. However, because GI does not permit the testing of foods with little or no carbohydrate, it cannot provide a guide to many foods containing protein and fat that are common components of everyday diet. Moreover, the GI methodology does not account for the insulin response. It has been widely assumed that the insulin response is proportional to the glucose response, and therefore the glycaemic response is an accurate predictor of the insulin response. However, this is not the case. For instance, as one increases the amount of a carbohydrate ingested, the amount of insulin does not increase proportionately. Studies with mixed meals have shown that the fat content of a mixed meal showed a significant inverse relation (*r* = − 0.60) with observed insulin responses and was a more reliable predictor of insulin demand than the amount of carbohydrate [35]. Although carbohydrate is the primary stimulus for insulin secretion, fat and protein content in the meals may also influence insulin release. As insulin captures the effects of a wider group of nutrients, it was chosen in the current study as a key serum marker.

Current research studies implicate both postprandial glycaemic and lipaemic responses in the development of cardiovascular disease and diabetes. However, they have been usually considered in isolation. Given the fact that carbohydrates and fats compete with clearance from the blood streams, gastro intestinal tract and peripheral cells, and their metabolism is always interrelated, for GlucoTRIG both insulin and lipaemic response was considered to the overall macronutrient composition of the meal. This may provide better insights into the relationship between the physiologic effects of foods/meals and their long term effects on health. Capturing the response of insulin and triglycerides to meals might provide an early indication of the healthiness of meals and provide more insights into physiological response than the glucose response alone. In a healthy adult consuming a healthy diet, serum insulin and triglyceride levels will have returned to a baseline level or closer to baseline levels in a certain time frame, following consumption of meal. It is argued that meals that lead to persistent high postprandial serum concentrations of insulin and triglycerides (i.e. remained elevated at the set time points) are unhealthy and most likely increasing the risk of development of type 2 diabetes and cardiovascular diseases.

### Rationale for post-prandial sampling time (180 min)

A 3 h (180 min) time point was chosen to capture the post-prandial lipid and insulin response of the meal. Conventionally, the glycaemic response is measured at 2 h, but this standard was established only as a diagnostic tool for identifying type 2 diabetes and impaired glucose tolerance, not to mark the total period of postprandial glucose elevation. Calculating a response for a greater time period can provide more physiological insights than a 2 h window period. Advances in understanding of key physiological mechanisms such as hepatic storage of glucose as glycogen, suppression of glucose release and insulin signalling, involved in the storage, maintenance and disposal of glucose after a mixed meal have provided insights into metabolism and normal energy expenditure in humans. A Study with ^13^C nuclear magnetic resonance spectroscopy [36] has shown complete suppression of hepatic glucose within 30 min post consumption of a mixed meal. However, this accounted for only a small proportion (28%) of the meal induced glucose incursions. Three to four hours post meal duration accounted for all of the plasma glucose and insulin changes, with a parallel increase in glycogen levels to maintain glucose homeostasis [36]. Hyperinsulinemia was shown to be a significant aetiological factor in obesity, type 2 diabetes, cardiovascular disorders, and neurodegenerative disorders [37]. Different insulin patterns was previously evaluated in response to a glucose load (100 g) administered over 3–5 h with plasma insulin levels assessed from baseline to 180 min [38]. With normal insulin tolerance, the peak insulin production occurs between 30 or 60 min levels, followed by a return to fasting range at 120 or 180 min [38]. Numerous studies whereby purified proteins have been fed demonstrate a sharp rise in insulin, rapid falling to baseline levels by 180 min [39, 40]. Studies have also shown that triglycerides peak at 3–4 h post meal consumption [24]. Patterns involving prolonged exposure of plasma insulin and failure to return to basal level were implicated in the development of insulin resistance and type 2 diabetes [41]. Based on the above discussed evidence, it is hypothesized that elevated serum insulin levels at 180 min post feeding (relative to baseline measures) and peak triglyceride levels at 180 min, are the indicators of an unhealthy metabolic state induced by diet.

### Single time point measurements vs area under the curve (AUC)

For GlucoTRIG, single time points for serum sampling have been selected, rather than determining the AUC of plasma concentrations across multiple time points post meal ingestion. The AUC methodology involves collection of multiple venous or capillary blood samples at multiple time points over a specific time period. Frequency of blood sampling and the duration of sampling (2 h in most cases) significantly influences the mean and variation for the resulting AUC values. Moreover, several methods are used to calculate the AUC (such as total AUC, incremental AUC), resulting in different dietary index values. For example, in the GI methodology, the AUC of plasma glucose concentration is calculated and expressed as a percentage of the area obtained after the ingestion of 50 g glucose/white bread. Lack of adhering to a standardised schedule of blood sampling with respect to both frequency and length of time likely affects the GI values, resulting in less consistent values [42]. We conjecture that use of an absolute value (difference between two time points) rather than AUC not only reduces the number of blood sample collections but can generate values with lower variation.

As serum insulin and triglycerides have different units (mmol and pmol) but the same base (Litre), and are interdependent (the rate of change of insulin can depend upon the rate of change of triglycerides or vice versa), multiplication was employed to combine them into a single factorial value over a specific time period (180 min) (GlucoTRIG) value. The final value for the respective test meal will be presented as a fraction of the GlucoTRIG value of the reference meal.

### The reference meal for GlucoTRIG

The advantage of using a reference food in a classification system is derived from the ability to test different meal groups. Between-subject variation can be diminished when the subjects’ individual responses are indexed to a reference meal. Testing the reference food more than two times reduces the effect of day-to-day variability within individuals. Hence, these sources of variation within and between subjects can be minimised to allow true differences to be detected among foods and meals in their capacity to stimulate glycaemic and lipaemic stimulations. The reference meal used in the current study, was tested three times with a minimum wash out period of 3 days. There were no statistically significant differences for the GlucoTRIG values of the reference meal between different days.

In the current study same amount of carbohydrate in the reference food as for GI (50 g). However, carbohydrates are not the only nutrient source to influence postprandial glycaemia. Other macronutrients in the diet, such as fat and protein, can influence the postprandial blood glucose level (e.g. adding dietary fat can slow glucose absorption, delaying the peak glycaemic response). High fat diets (approximately 60% of the energy from fat) have been shown to significantly influence the triglycerides peak and AUC. Postprandial studies report a greater increase in total plasma triglyceride levels after a high fat meal (about 68 g total fat) compared to a moderate fat meal (about 35 g total fat) [43]. Moderate fat diets (approximately 30–40% of the energy intake from fat, typically containing monounsaturated fats and saturated fat) are reflective of more typical dietary behaviour patterns. The moderate fat intake levels in the reference meal reflects fat levels in typical diets.

### Limitations

This study represents a first attempt to factor in both glycaemic and lipaemic responses collectively to evaluate the healthiness of mixed meals and foods, GlucoTRIG. This study is primarily focused on description of the methodology and reference food for future testing of foods for testing GlucoTRIG. Further studies are warranted to establish the efficacy of GlucoTRIG as a health indicator of foods by assessing GlucoTRIG values of the isocaloric meals and foods differing in macronutrient composition. This study although conducted with adequate sample size, does not allow sex-based stratification of the GlucoTRIG values sex-sensitive approach might be worth considering for future testing of GlucoTRIG for categorising healthiness of meals and foods. Previous studies on post-prandial methodology for healthiness indicators of foods such as GI has indicated inclusion of 10 subjects provide enough power and reasonable precision for measuring the foods based response. However, increase in the number of subjects might be worth considering for detecting small GlucoTRIG related based differences, sex sensitive differences or to increase the precision of the GlucoTRIG values.

## Conclusion

In the current study, an novel index and a reference standard for classifying meals was described based on their glycaemic and lipaemic responses with the objective of assessing their overall healthiness. The macronutrient composition of foods undoubtedly makes a major contribution to predicting the GlucoTRIG value, however there could be several other factors such as rate of starch digestion, food structure, rate of release of lipids, the amount of rapidly available sugars, fibre, and biological factors such as rate of gastric emptying that also influence the GlucoTRIG value. Further research is required to determine the relationship between GlucoTRIG and other factors to improve its predictability and applicability to a wide range of foods. It is also important to test GlucoTRIG as a predictor of early stage disease development. GlucoTRIG applies to healthy individuals and the usefulness of GlucoTRIG needs further testing in individuals with insulin resistance or compromised β-cell function. Nonetheless, the concept may be relevant in the early stages of disease development, when β cell function is still adequate for preventing the development of epidemic diseases such as obesity, type 2 diabetes and cardiovascular disease**.**

## Data Availability

The datasets used or analysed during the current study are available from the corresponding author on reasonable request.

## Abbreviations

GI: Glycaemic index
BMI: Body mass index
AUC: Area under the curve
